# How Does Organizational Career Management Benefit Employees? The Impact of the “Enabling” and “Energizing” Paths of Organizational Career Management on Employability and Job Burnout

**DOI:** 10.3390/ijerph20021259

**Published:** 2023-01-10

**Authors:** Mengying Xie, Guorui Wang, Yenchun Jim Wu, Haohua Shi

**Affiliations:** 1 Faculty of Business Administration, School of Business Administration, Southwestern University of Finance and Economics, Chengdu 611130, China; 2 Graduate Institute of Global Business and Strategy, National Taiwan Normal University, Taipei 106, Taiwan; 3 Department of Hospitality Management, Ming Chuan University, Taipei 111, Taiwan

**Keywords:** organizational career management, learning, vitality, self-perceived employability, job burnout, career plateau

## Abstract

Organizational career management (OCM) is believed to be a useful practice to stimulate the potential of employees. However, how this can be achieved is still under investigation. This research aims to explore the mechanisms that explain the effects of OCM by clarifying its impact on employees’ psychological states and their capability, based on a socially embedded model of thriving. To examine our hypotheses, we conducted a three-wave survey study with 272 full-time employees in China from diverse industries. The study lasted for three months and there was a one-month interval after each wave. We asked the participants to report OCM, career plateau and demographic variables at Time 1, their appraisal of learning and vitality at Time 2, and their self-perceived employability and job burnout at Time 3. We utilized regression analysis to examine our theoretical model and path analysis using the bias-corrected bootstrap method to test the significance of the indirect and moderation effects. The findings showed that OCM positively affected employees’ learning and vitality at work, which increased their self-perceived employability and subsequently decreased job burnout. Furthermore, the effects of OCM were found to be weaker for employees with a high degree of career plateau. These findings demonstrate that OCM benefits employees by “enabling” and “energizing” them to better themselves in terms of their employment and they shed light on the boundary condition of the career plateau. Therefore, organizations may provide OCM to facilitate employees’ capability and their motivation to engage in self-development, and to further enhance the effects by decreasing their perception of a career plateau.

## 1. Introduction

Organizational career management (OCM), which is defined as “the organization carrying out activities relevant to the career development of its employees” [1], can stimulate employee potential and help employees to explore their personal career goals [2,3]. In recent years, OCM has received increasing attention from scholars and practitioners [4,5]. It has been found that OCM can increase the potential of employees, help to retain employees and promote their self-realization [6,7], increase career success [8], and reduce turnover intention [9].

Previous research on OCM has resulted in useful knowledge of its benefits, as summarized above (e.g., employees’ self-realization and intention to maintain employment); however, how OCM affects employees’ capabilities and psychological states has received little attention [8]. To investigate this question, we drew upon a socially embedded model of thriving at work [10], which illustrates how the employee’s experience changes when they are embedded in a work context that favors self-development, for example, it provides opportunities for training, coaching, and mentoring. The socially embedded model demonstrates that employees in such a work context report that they thrive at work, which includes experiencing learning as a positive cognitive experience and experiencing vitality as a positive psychological feeling. These positive experiences of learning and vitality lead to capability development and psychological well-being [10,11]. 

Drawing on the socially embedded model, we suggest that OCM functions as a favorable work context for self-development, which may help employees better adapt to their job in terms of their cognition and psychology. On the one hand, from a cognitive viewpoint, a variety of policies, such as training via OCM, “enable” employees’ abilities and skills so that employees are better suited to jobs, thus increasing employees’ “employability” [12]. On the other hand, from an affective perspective, OCM “energizes” employees by attracting and motivating them in exchange for their ongoing commitment and hard work [13]. These effects can help to energize employees during the workflow process and to further decrease employees’ job burnout. Therefore, the first aim of this study was to examine whether OCM can “enable” employees through career employability and “energize” employees in order to achieve low levels of job burnout.

Furthermore, given the mechanism of thriving provided by the socially embedded model, we attempted to reveal how OCM “enables” and “energizes” employees. As an important psychological state of employees, thriving at work implies that employees simultaneously experience the cognitive state of “learning” and the affective state of “vitality”, which promote their growth and development [11,14]. Accordingly, we integrated learning/vitality into our hypothesized model, and explored how OCM influences employees’ employability and job burnout. We expected that, on the one hand, OCM would facilitate the “learning” state of employees, enabling employees to acquire more knowledge, experience, and skills to complete work tasks, and increase employees’ employability; on the other hand, we expected that OCM would motivate employees by energizing them with “vitality” at work, helping them maintain a positive mood in the workplace, which further decreases employee burnout.

**Figure 1:**
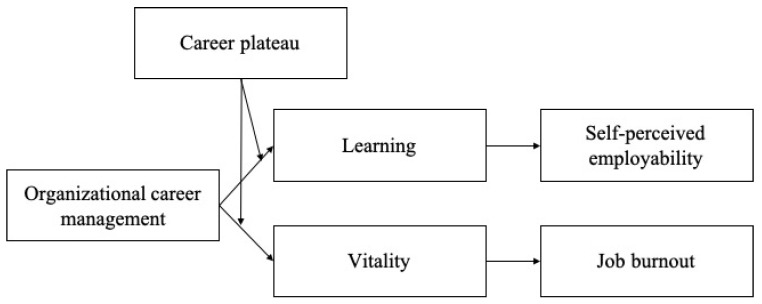
Theoretical model.

However, we believe that the influence of OCM on employees depends on each individual’s perceived career plateau, which is defined as the “stagnation of an individual’s career development in the current organization” [15] (p. 602). Although OCM benefits employees by increasing their capabilities and energy, it has a weaker effect if employees perceive a high degree of career plateau [16]. Thus, the final aim of the present study was to examine how career plateaus influence the relationship between OCM and thriving. Our full model is depicted in Figure 1.

## 2. Theory and Hypothesis

OCM is a set of management practices that are implemented by organizations to promote the achievement of employees’ career development goals [17]. It mainly consists of four dimensions: fairness in promotion, emphasis on training, communication of career development information, and career self-awareness [5]. In this study, we expected OCM to benefit employees by stimulating the “enabling” path and the “energizing” path.

### 2.1. The “Enabling” Path

#### 2.1.1. Organizational Career Management and Self-Perceived Employability

Vanhercke et al. [18] defined perceived employability as an individual’s perception of his or her own ability to obtain and maintain their current employment (as well as to obtain new employment). It concerns an individual’s perception of their ability to obtain and maintain employment within an organization and their chance of employment in the job market [19]. We expected that OCM provided by an organization, such as in the form of training, would promote employees’ self-perceived employability [20]. First, an emphasis on training can be viewed as a job resource because it can help employees enhance their potential and develop personal skills through various management activities [21], which in turn, increase employees’ cognition of their employability. Second, by offering a fairly transparent promotion system and information on organizational job vacancies, OCM can help employees deal with the challenges of job demands and withstand pressure to adapt to the current work environment [22]. Third, by providing career information and other career management activities, organizations can motivate employees to recognize their own abilities, thus leading to improved employability. Therefore, we proposed the following:

**Hypothesis 1** **(H1).** *Organizational career management is positively related to employees’ self-perceived employability.*

#### 2.1.2. The Mediating Role of Learning

According to a socially embedded model, a work context characterized by favorable unit features and resources (e.g., promotion opportunities, training, coaching, and mentoring for personal development) for employees to achieve personal growth may motivate employees to thrive at work [10]. Spreitzer et al. [10] theorized workplace thriving as the states of learning and vitality that are experienced by employees. Learning refers to acquiring knowledge and experience from the processes of interacting with, communicating with, and observing and imitating others [23].

We expected the “enabling” path to be stimulated by the mediating role of learning. OCM can be seen as unit contextual features that provide opportunities and career information and emphasize the importance of learning, which in turn, increase an employee’s learning state [24]. Specifically, on the one hand, the training and self-career awareness education included in OCM provides employees with a variety of opportunities for learning [25]. On the other hand, OCM can also emphasize the importance of improving one’s ability through fair and transparent promotion, building clear employee self-awareness [26] so that the expectation of positive outcomes, such as promotions, motivates them to learn.

Furthermore, based on a socially embedded model of thriving, learning can promote individual development [27], that is, with continued learning, individuals make great progress in regard to their developmental processes (e.g., self-perceived employability). We proposed that learning acts as an important catalyst for employees’ self-perceived employability by developing greater competency, facilitating acute observation, and maintaining confidence. First, when employees learn and grow at work, they develop competencies inside and outside the organization, along with increased knowledge and promotion abilities [14]. Second, the understanding and accumulation of knowledge will promote employees’ acute observational abilities, making it possible for them to obtain more information regarding better jobs [28]. Thus, we hypothesized the following:

**Hypothesis 2** **(H2).** *Learning mediates the relationship between organizational career management and employees’ self-perceived employability.*

### 2.2. The “Energizing” Path

#### 2.2.1. Organizational Career Management and Job Burnout

In this study, we expected OCM to not only influence the individual’s cognition of their employability but to also impact employees’ job burnout in an affective way. Burnout is a state of physical and mental exhaustion that presents after working for a long period of time [29]. Building on previous studies, we proposed that OCM decreases individuals’ job burnout by maintaining positive affectivity and facilitating resource accumulation.

On the one hand, OCM can encourage employees to perceive that organizations care about their employees’ development [24]. Such feelings stimulate positive emotions in employees, which reduces negative affectivity, such as feelings of boredom and fatigue [30], thus decreasing their burnout. On the other hand, OCM helps employees build resources that are important to them, such as psychological resources (which help employees to become more optimistic about their future) [20]. Therefore, when employees experience a high level of OCM, the resulting increase in career self-knowledge in relation to their personal interests and goals can increase employees’ career safety perceptions and decrease employee burnout. Thus, we hypothesized the following:

**Hypothesis 3** **(H3).** *Organizational career management is negatively related to employee burnout.*

#### 2.2.2. The Mediating Role of Vitality

In addition to the role of the “enabling” path of OCM in increasing employee abilities, we also expected OCM to “energize” employees by triggering employees’ vitality in the workplace. Vitality is the emotional component of thriving and it represents the state of energy and enthusiasm of employees [11]. Similar to learning, we proposed that OCM is also an important indicator of vitality. Specifically, OCM can increase career identity, which describes an individual’s awareness of their personal interests and goals [31]. It helps employees realize their strengths and opportunities by focusing on their tasks, which induces employee vitality in the workplace. Additionally, OCM can also increase employees’ career confidence with regard to the future, which promotes work meaningfulness and enthusiasm for their work [32].

Moreover, we expected that vitality may reduce job burnout. According to a socially embedded model of thriving, when filled with vitality, employees will perceive themselves as having greater fuel and energy, which helps them to avoid negative affectivity (e.g., boredom and burnout) [14]. Moreover, when employees experience positive emotions, such as vitality, they will enjoy their work time and be immersed in their work [33], and this “happy time” helps individuals avoid burnout. Taken together, we hypothesized the following:

**Hypothesis 4** **(H4).** *Vitality mediates the relationship between organizational career management and employee burnout.*

### 2.3. The Moderating Role of Career Plateaus

We next considered how OCM interacts with career plateaus to affect employees’ learning/vitality. Xie and Long [34] defined the career plateau as the phenomenon of an individual’s stagnation in their current organization. Employees on a plateau are less satisfied with their supervisors, report fewer opportunities for advancement, are absent more often, rate their employability in the market as lower, and feel less healthy [35]. We proposed that the career plateau plays a moderating role in the relationship between OCM and employee learning/vitality. Specifically, a high degree of career plateau corresponds to fewer opportunities to learn new things and less likelihood of developing personal skills, thus weakening the positive effect of OCM on the perception of the learning state in the current organization. In addition, when employees have a high degree of career plateau, they increasingly experience their job as having few challenges and little excitement and enthusiasm [36]. These negative emotions and experiences inhibit employees’ perception of a vitality state, even though they receive a high level of OCM [37].

In contrast, for employees with a low degree of career plateau, OCM offers sufficient vocational training, fair and open promotion channels, and clear career planning. These policies enable employees to find learning opportunities [38], and thus they can devote themselves to their work and maintain a high motivational state. In addition, OCM can motivate employees to learn and maintain vitality by providing large amounts of information that can easily be obtained. Thus, we proposed the following:

**Hypothesis 5** **(H5).** *Career plateaus moderate the relationship between OCM and learning such that when employees experience a higher degree of career plateau, the relationship between career management and learning is weaker.*

**Hypothesis 6** **(H6).** *Career plateaus moderate the relationship between OCM and vitality such that when employees experience a higher degree of career plateau, the relationship between career management and vitality is weaker.*

## 3. Method

### 3.1. Participants and Procedure

In this paper, the snowball sampling method was used to conduct a longitudinal survey. Through online survey websites, we collected data from full-time employees in China working in a wide range of industries such as education, banking, and the internet sectors. Using a longitudinal design, the data were obtained in three waves, with a one-month interval after each wave to minimize potential common method variance concerns [39]. For the first wave, 380 questionnaires were collected. One month later, 310 questionnaires were matched in the second collection, and the response rate was 81.60%. After a further month, 285 questionnaires were matched in the third collection, and the response rate was 75%. Finally, excluding the 13 employees who did not pass the attention checks, a total of 272 samples were selected.

The three-month test period was divided into three periods. In the first month, OCM, career plateau, and demographic variable data were collected; in the second month, thriving (learning and vitality) data were collected; and in the third month, self-perceived employability and job burnout data were collected. The participant demographics were as follows: 50% were female, the average age was 36.88 years, the average tenure in their current organization was 12.43 years, and 63% had a Bachelor’s degree.

### 3.2. Measures

We employed existing measures that were published in high-quality journals and generally used in empirical research to evaluate all of the variables in the theoretical model. Since the participants were Chinese speakers, the questionnaire was in Chinese, and we translated the items from English into Chinese following the translation–back translation procedure suggested by Brislin [40]. All measures were scored on a 5-point scale ranging from 1 (completely disagree) to 5 (completely agree).

Organizational career management. We measured employees’ perceptions of organizational career management with 16 items developed by Long et al. [5]. For example, one item was “My current company can provide promotion path information”. Cronbach’s alpha for the scale was 0.94.

Learning. We measured learning with the scale of thriving developed by Porath et al. [14]. The scale of thriving includes two dimensions: learning and vitality. The learning dimension uses 5 items to evaluate whether employees feel that they are acquiring and are able to apply valuable knowledge and skills. For example, one of the items regarding learning was “I felt that I was constantly improving”. Cronbach’s alpha was 0.74 for learning.

Vitality. We evaluated the vitality of employees by the vitality dimension in the scale of thriving [14]. The vitality dimension in this scale uses 5 items to appraise employees’ positive feelings in relation to having energy available and feeling “alive”, for example, “I was full of energy”. Cronbach’s alpha was 0.75 for vitality.

Job burnout. To evaluate the job burnout of employees, we used the scale of experienced burnout developed by Maslach and Jackson [29], which included items such as “I feel emotionally drained from my work”. Cronbach’s alpha for the scale was 0.89.

Self-perceived employability. Self-perceived employability was measured using the method of Rothwell and Arnold [41] and an 11-item scale. This included items such as “I have good prospects in this organization because my employer values my personal contribution”. Cronbach’s alpha for the scale was 0.94.

Career plateau. We measured career plateau with 16 items adapted from Xie and Long [34], for example, one item was “My current job requires me to keep learning new things”. Cronbach’s alpha for the scale was 0.86.

### 3.3. Analytical Strategy

We used regression analysis to examine our theoretical model with Mplus 7.0 [42]. We further conducted a bias-corrected bootstrap analysis to test the significance of indirect effects in the path analysis using 5000 bootstrap samples. Moreover, we used a moderated path analysis approach adopted by Edwards and Lambert [43] to test the first-stage moderating effect.

### 3.4. Results

#### 3.4.1. Confirmatory Factor Analyses

The CFA results are shown in Table 1. An analysis of the measurement model indicated that the six-factor model fitted the data well, with χ^2^ (*N* = 272) = 2163.97, SRMR = 0.05, RMSEA = 0.05, CFI = 0.91 and TLI = 0.90, and it was better than alternative models.

#### 3.4.2. Descriptive Results

The means, standard deviations, and correlations are presented in Table 2. Organizational career management was positively associated with learning (*r* = 0.31, *p* < 0.01) and vitality (*r* = 0.44, *p* < 0.01); employees’ learning was positively related to self-perceived employability (*r* = 0.44, *p* < 0.01); and employees’ vitality was negatively associated with job burnout (*r* = −0.51, *p* < 0.01). The results initially verified our hypotheses.

#### 3.4.3. Hypothesis Tests

**Figure 2:**
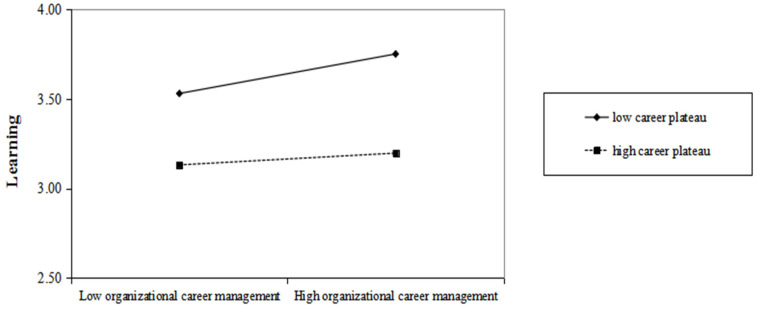
Moderating role of career plateau on organizational career management and employee learning.

**Figure 3:**
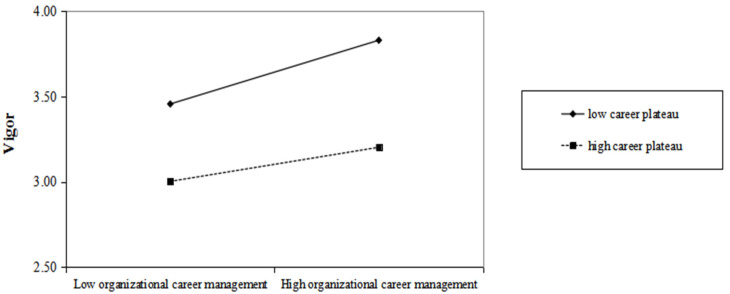
Moderating role of career plateau on organizational career management and employee vitality.

Table 3 shows the results of our path analyses. Figure 2 and Figure 3 plot the moderating effect of the career plateau.

The “enabling” path. Hypothesis 1 indicated that organizational career management was positively related to self-perceived employability. As is shown in Table 3, the results revealed that organizational career management was significantly and positively associated with self-perceived employability (*β* = 0.47, *p* < 0.001), supporting hypothesis 1. Hypothesis 2 posited that learning had a mediating role in the relationship between OCM and self-perceived employability. As is shown in Table 3, OCM was significantly and positively related to learning (*β* = 0.33, *p* < 0.001), and learning was significantly and positively associated with self-perceived employability (*β* = 0.20, *p* < 0.01). Moreover, the indirect effect was 0.08, and the 95% bias-corrected confidence interval was [0.025, 0.129]. Thus, hypothesis 2 was supported.

The “energized” path. Hypothesis 3 indicated that OCM was negatively related to job burnout. As shown in Table 3, the results indicated that OCM was significantly and negatively associated with job burnout (*β* = −0.29, *p* < 0.001), supporting hypothesis 3. Hypothesis 4 suggested that vitality had a mediating role in the relationship between OCM and job burnout. As is shown in Table 3, OCM was significantly and positively related to vitality (*β* = 0.49, *p* < 0.001), and vitality was negatively related to job burnout (*β* = −0.29, *p* < 0.001). In addition, the indirect effect was −0.15, and the 95% bias-corrected confidence interval was [−0.223, −0.068]. Thus, hypothesis 4 was supported.

Moderating effect. Hypothesis 5 suggested that career plateaus moderate the relationship between OCM and learning such that when employees experience a higher degree of career plateau, the relationship between career management and learning is weaker. As is shown in Table 3 and Figure 3, the interaction between OCM and career plateau significantly and negatively affected learning (*β* = −0.14, *p* < 0.05), supporting hypothesis 5. Similarly, hypothesis 6 suggested that the career plateau moderates the relationship between OCM and vitality such that when employees experience a higher degree of career plateau, the relationship between career management and vitality is weaker. As is shown in Table 3, the interaction between OCM and career plateau significantly and negatively affected vitality (*β* = −0.16, *p* < 0.05), supporting hypothesis 6.

## 4. Discussion

The current study represents an attempt to use a socially embedded model of thriving at work to explain the effects of OCM in organizations. We found that OCM triggers a high level of self-perceived employability and a low level of job burnout via learning and vitality, respectively. Furthermore, we found that career plateaus moderated the effect of OCM on learning/vitality such that this effect was weaker among those who experienced higher (vs. lower) degrees of career plateau.

### 4.1. Theoretical Contribution

Our research makes a number of theoretical contributions. First, the current findings provide support for the mechanisms suggested by using a socially embedded model of thriving at work. Although some studies have proposed that organizational support behavior and manager support can improve self-perceived employability and decrease burnout [8], they did not empirically study how OCM affects employees’ capabilities and psychological states. The current study proves that OCM has significant positive effects on self-perceived employability and negative effects on burnout. This means that the promotion system and information, career development information, developmental training, and management measures to promote career self-cognition provided by the company have a positive impact on learning [25] and maintaining vitality [4]. To some extent, these findings explain how the effects of OCM are transmitted to impact employees’ self-perceived employability and job burnout.

Second, we have contributed to the research on OCM by investigating learning/vitality as two meditating mechanisms that explain the relationship between OCM and self-perceived employability/job burnout. This approach enabled the current research to examine the “enabling path” and “energizing path”—when organizations implement OCM policies, employees may not only increase their personal skills and abilities [44], but also become more energetic at work. This complements previous studies that have linked both the “enabling path” and the “energizing path”. Based on a socially embedded model of thriving at work, on the one hand, OCM enables employees to acquire more knowledge and develop their personal skills through the cognitive state of learning [45], which eventually increases their employability. On the other hand, OCM energizes employees to maintain their vitality and dedication to their work in the workplace [46], which decreases their feelings of burnout. As such, this research further supplements research on the consequences of OCM by exploring the effects of learning/vitality on employees’ self-perceived employability/job burnout.

Third, we verified that career plateaus play a moderating role in the relationship between OCM and learning/vitality. This is another important theoretical contribution of this study, which not only enriches the theories related to the knowledge of management but also expands the application of OCM in a specific context. The findings imply that a high degree of career plateau diminishes the positive effects of OCM, and employees with a high degree of career plateau may be susceptible to the negative effects of the dilemma of career development [47], causing them to undertake fewer learning activities and maintain a low state of vitality. As a consequence, although organizations may implement OCM policies, these employees will have lower self-perceived employability and greater job burnout.

### 4.2. Practical Implications

The current research has several important implications for organizational management. First, the findings of our study suggest that due to its benefits, organizations and managers should pay more attention to OCM [48]. For example, the application of OCM will improve individuals’ employability and reduce job burnout [49]. Specifically, when there is an emphasis on training and development, employees will make clearer career plans; when there are opportunities for job mobility, employees can achieve their career ambitions; and if career self-awareness is specified, employees can find advantages and acquire more knowledge.

Second, this study also found that learning/vitality mediates the relationship between OCM and employees’ self-perceived employability and job burnout. That is, managers should take appropriate measures to help employees maintain a state of high energy and continuous acquisition of knowledge and skills so that employees mobilize their energy to work in all aspects and continue to thrive.

Third, to prevent employee career plateaus, organizations and managers should use various job designs to prevent employee perceptions of stagnation in their current organization. One example is job enlargement, wherein qualitatively comparable tasks are assigned to an employee [50], for instance, additional projects in similar areas. In addition, organizations and managers should design multiple career development paths for employees [51].

## 5. Conclusions

The current study represents an attempt to use a socially embedded model of thriving at work to explain the effects of OCM in organizations. We found that OCM triggers a high level of self-perceived employability and a low level of job burnout via learning and vitality, respectively. Furthermore, we found that career plateaus moderated the effects of OCM on learning/vitality such that these effects were weaker among those who experienced higher degrees of career plateau.

Our research makes a number of theoretical contributions. First, the current findings provide support for the mechanisms suggested by using a socially embedded model of thriving at work. Previous studies have not empirically studied how OCM affects employees’ capabilities and psychological states. Our findings clarified that the influence of OCM has a positive impact on learning and maintaining vitality. Second, we have contributed to research on OCM by investigating the “enabling path” and “energizing path”. As such, this research further supplements research on the consequences of OCM by further exploring the effects of learning/vitality on employees’ self-perceived employability/job burnout. Third, we verified that career plateaus play a moderating role in the relationship between OCM and learning/vitality. This argument not only enriches the theories related to the knowledge of management but also expands the application of OCM in specific contexts. The findings imply that a high degree of career plateau diminishes the positive effect of OCM, and employees with a high degree of career plateau may be susceptible to the negative effects of the dilemma of career development, causing them to undertake fewer learning activities and maintain a low level of vitality.

It should be noted that this study also has some limitations and shortcomings. First, the current study used a time-lagged design to examine the effects of OCM. We were not able to confirm causal effects, and as a consequence, the relationships between OCM and employee capabilities and psychological states cannot be confirmed. It is suggested that future studies adopt a longitudinal design to fill this gap. Second, we adopted the self-reporting method to measure variables, which may have led to common method bias. The results of Harman’s single factor tests suggested that the first factor was below 26.13%, indicating that common method bias was not a serious problem in the current paper. In the future, a multi-source data collection method is suggested. Third, consistent with previous studies, we focused on the moderating effects of career plateaus. However, temporary “stagnation” in career development may have a bright side. When experiencing a career plateau, individuals can re-examine and plan their future career development, which may also help them to start a new career journey. We suggest that future research should examine the benefits of career plateaus.

## Figures and Tables

**Table 1:** Confirmatory factor analysis.

Model	χ^2^	df	$\chi^{2}/df$	SRMR	RMSEA	CFI	TLI
Six-factor model	2163.97	1341	1.61	0.05	0.05	0.91	0.90
Five-factor model	3026.51	1367	2.21	0.06	0.07	0.82	0.81
Four-factor model	3786.02	1371	2.76	0.09	0.08	0.73	0.72
Three-factor model	4386.42	1374	3.19	0.10	0.09	0.66	0.65
Two-factor model	5784.20	1376	4.20	0.13	0.11	0.51	0.49
One-factor model	6296.58	1377	4.57	0.13	0.12	0.45	0.43

Note. Six-factor model: organizational career management, career plateau, learning, vitality, self-perceived employability, job burnout; Five-factor model: organizational career management, career plateau, learning + vitality, self-perceived employability, job burnout; Four-factor model: organizational career management + career plateau, learning + vitality, self-perceived employability, job burnout; Three-factor model: organizational career management + career plateau + learning + vitality, self-perceived employability, job burnout; Two-factor model: organizational career management + career plateau + learning + vitality, self-perceived employability + job burnout; One-factor model: organizational career management + career plateau + learning + vitality + self-perceived employability + job burnout.

**Table 2:** Descriptive statistics and correlations of current study.

Variable	M	SD	1	2	3	4	5	6	7	8	9	10	11
1. Gender	1.50	0.50	1										
2. Age	37.71	10.37	0.08	1									
3. Education	1.89	0.65	0.11	−0.39 **	1								
4. Work years	15.80	10.04	0.05	0.82 **	−0.42 **	1							
5. Work years in current company	12.43	9.88	0.09	0.74 **	−0.34 **	0.85 **	1						
6. Organizational career management (T1)	3.59	0.56	−0.13 *	−0.12 *	−0.03	−0.09	−0.10	(0.94)					
7. Career plateau (T1)	2.69	0.50	0.21 **	0.09	−0.12 *	0.10	0.11	−0.57 **	(0.86)				
8. Learning (T2)	3.76	0.58	−0.12 *	0.05	0.09	0.07	0.03	0.31 **	−0.46 **	(0.74)			
9. Vitality (T2)	3.56	0.59	−0.06	0.05	0.01	0.10	0.10	0.44 **	−0.54 **	0.72 **	(0.75)		
10. Self-perceived employability (T3)	3.41	0.59	−0.22 **	−0.05	0.05	−0.04	−0.08	0.46 **	−0.68 **	0.44 **	0.46 **	(0.94)	
11. Job burnout(T3)	2.56	0.51	0.04	−0.12 *	0.07	−0.15 *	−0.16 **	−0.30 **	0.39 **	−0.44 **	−0.51 **	−0.25 **	(0.89)

Note. *N* = 272. Gender was coded 1 = male, 2 = female. Reliabilities are reported along the diagonal where applicable. * *p* < 0.05. ** *p* < 0.01.

**Table 3:** Results of regression analysis.

Predictors	Learning		Vitality		Self-Perceived Employability		Job Burnout	
Model	M1	M2	M3	M4	M5	M6	M7	M8
Intercept	2.16 ***	3.40 ***	1.56 ***	3.37 ***	1.71 ***	1.01 **	3.79 ***	4.56 ***
Gender	−0.13 *	−0.06	−0.03	0.05	−0.20 **	−0.17 **	0.02	−0.01
Age	0.004	0.002	−0.001	−0.004	0.004	0.003	−0.002	−0.001
Education	0.18 **	0.11 *	0.09	0.02	0.10	0.05	−0.01	0.04
Work years	0.01	0.01	0.01	0.01	0.01	0.002	−0.003	0.001
Work years in current company	−0.01	−0.003	0.01	0.01	−0.01	−0.01	−0.01	−0.01
Organizational career management	0.33 ***	0.13	0.49 ***	0.26 **	0.47 ***	0.32 ***	−0.29 ***	−0.10
Learning						0.20 **		−0.15 *
Vitality						0.18 *		−0.29 ***
Career plateau		−0.48 ***		−0.54 ***				
Organizational career management × Career plateau		−0.14 *		−0.16 *				
*R* ^2^	0.14 ***	0.26 ***	0.22 ***	0.36 ***	0.25 ***	0.32 ***	0.13 **	0.25 ***

Note. *N* = 272. * *p* < 0.05. ** *p* < 0.01. *** *p* < 0.001.

## Data Availability

Due to the nature of this research, participants in this study did not agree to their data being shared publicly, so supporting data are not available.
